# New Treatment for Pyorrhœa Alveolaris

**Published:** 1896-04

**Authors:** C. H. Rosenthal


### New Treatment for Pyorrhœa Alveolaris.

In presenting a treatment for Pyorrhœa Alveolaris I will not attempt to give a scientific basis for my method, since it was conceived of observation rather than of profound scientific research.

Several months ago I had occasion to see a mouth in which I had placed a piece of bridge-work three months prior. The bridge consisted of two gold caps on the lower cuspid teeth, sustaining the central and lateral incisors which, as a result of pyorrhea alviolaris, had been lost. The cuspids to which the crowns were attached, as well as the approximal bicuspids, were also badly affected; so much so, in fact, that at the time I was apprehensive of the result. To my astonishment, three months after the operation, I found that the cuspid teeth had regained much of their firmness and an entire cessation of secretions. The bicuspids were still in the same condition as when the work was done. This caused me to suspect that the presence of the metal, which was driven well under the free mergins of the gum and made of 20-karat gold, alloyed with silver only, might account for the cure. I at once placed gold bands around the necks of the bicuspid teeth, cementing them firmly in place to prevent riding. In an incredibly short time about these teetb, too, the flow of pus stopped. I have tried this method in three cases since with uniform by good results. In the most recent case, instead of using the gold bands, I used pure silver. This idea was suggested recently at the Johns Hopkins University, where experiments were made on silver disks by pouring pus cultures on glass slabs; where the cultures came in contact with the silver they at once became innocuous. This silver-band experiment proved by far the most valuable. In the short time of five days there was an entire abatement of the secretions. This led me to believe that it was the silver contained in the gold crown of my first experiments that did the work. The experiments at the Johns Hopkins University shows silver to be the best agent of all the metals to destroy pus cultures.

The method of adjusting the bands is much the same as making a gold crown—fitting snugly to the tooth and cementing firmly. None of the metal need be exposed to view, since the only object is to have it in contact with the diseased tissue. I have not removed the bands in any of the cases, and therefore cannot state if there will be a recurrence of the trouble. I am of opinion, however, that if all the teeth affected were treated in this manner, and the disease entirely eradicated from the mouth, there would be no recurrence. Should this not prove true the bands left on the teeth permanently would certainly be an improvement on pyorrhœa alveolaris. More anon.

C. H. Rosenthal, D.D.S.

